# Retrograde Transpubic Approach for Percutaneous Radiofrequency Ablation and Cementoplasty of Acetabular Metastasis

**DOI:** 10.1155/2015/146963

**Published:** 2015-09-29

**Authors:** Salem Bauones, Veronique Freire, Thomas P. Moser

**Affiliations:** Department of Radiology, Hôpital Saint-Luc, Centre Hospitalier de l'Université de Montréal (CHUM), 1058 rue Saint-Denis, Montreal, QC, Canada H2X 3J4

## Abstract

We report a case of painful and disabling anterior acetabular bone metastasis treated with bipolar radiofrequency ablation and cementoplasty. Due to the high risk of complications related to the proximity of the femoral neurovascular structures with a direct approach, we successfully performed a retrograde transpubic approach under combined CT and fluoroscopic guidance. In the present report, we describe this approach detailing its indications, advantages, and the technical tips to achieve a safe and satisfactory procedure.

## 1. Introduction

Bone metastasis, particularly if osteolytic and in a weight-bearing bone, is associated with potential disabling complications, sometimes early in the course of the disease [1].

Image-guided percutaneous tumor management has been successfully used in the treatment of localized bone metastasis and its local complications. Different image-guided percutaneous interventional techniques are now available, including cementoplasty, chemical and thermal ablation, coblation, irreversible electroporation, and high intensity focused ultrasound [2, 3]. Such minimally invasive techniques aim to provide analgesic effect through tumor destruction and/or mechanical support for weight-bearing bones [4]. Indeed, cementoplasty and thermal ablation of bone tumor have evolved to become established as an effective palliative treatment of focal bone metastasis [5, 6].

Pelvic bone and particularly the acetabulum are frequently involved by bone metastases. However, their percutaneous treatment is sometimes hampered by technical difficulties related to a complex anatomy and the proximity of critical structures such as nerves, iliac or femoral vessels, and pelvic organs [7].

Hence, we aimed to report a case of an anterior acetabular metastasis treated by bipolar radiofrequency (RF) ablation followed by cementoplasty through a retrograde transpubic approach to avoid potential complications of a direct approach. This approach has not been previously described in the radiological literature and could be helpful for treating complex cases of pelvic bone metastasis.

## 2. Case Report

A 60-year-old female with a history of metastatic lung cancer treated with chemotherapy and radiotherapy was referred for significant left hip pain (Visual Analogue Scale score 90/100 mm). It was attributed to an osteolytic bone metastasis involving the left anterior acetabulum and considered at high risk for pathologic fracture.

Unfortunately, the medical treatment with opioid analgesics and bisphosphonates, which was tried for more than 2 months, was ineffective in improving the patient's pain and limp. On the other hand, the patient refused radiation therapy and a surgical treatment was not indicated because of the multiple metastases, associated comorbidities, and short life expectancy. Therefore, a palliative treatment combining percutaneous RF ablation and cementoplasty of the acetabular lesion was offered after oncologic interdisciplinary discussion.

**Figure 1:**
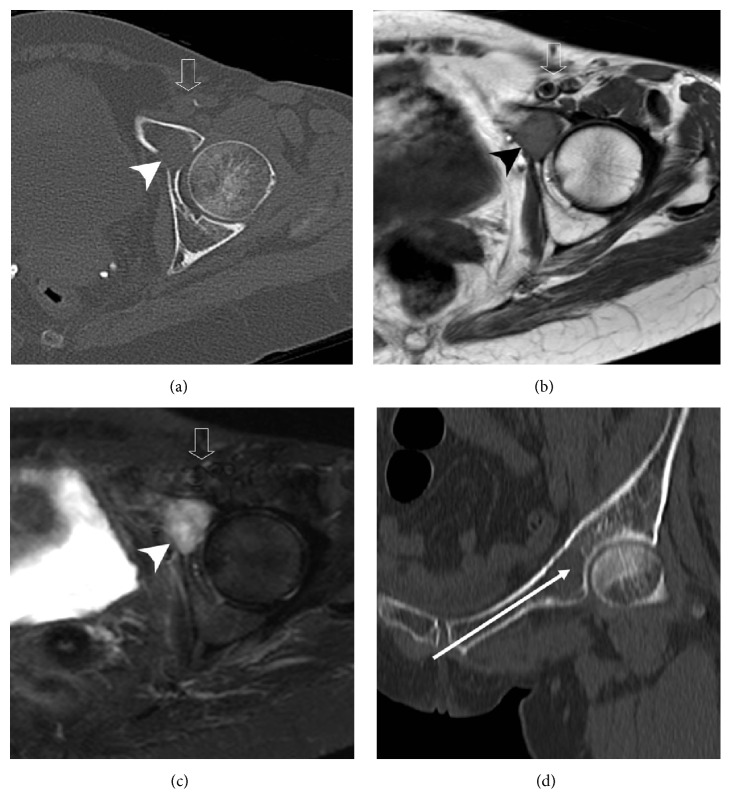
60-year-old-female with metastatic lung cancer complaining of left hip pain. (a) Nonenhanced axial CT image demonstrates left anterior acetabular osteolytic lesion (arrowhead) with anterior cortical thinning and posterior cortical breach. (b) and (c) The acetabular lesion (arrowhead) shows low signal intensity on axial T1W image (b) and high signal intensity on axial STIR image (c). Note its close relationship with the femoral neurovascular structures precluding a direct anterior approach (open arrow). (d) Coronal oblique reformatted CT image shows the needle path from the skin entry to the target lesion (long arrow).

A preoperative radiologic assessment was performed with computed tomography (CT) and magnetic resonance (MR) studies. The CT-scan demonstrated a geographic slightly expansile osteolytic lesion, measuring 2.5 × 2 cm in its axial plane and involving the left anterior acetabular column and proximal aspect of the superior pubic ramus, with anterior cortical thinning and posterior and lateral cortical breaches communicating with the hip joint (Figure 1(a)). On MR images, the lesion showed low signal intensity on T1-weighted images and high signal intensity on T2-weighted images (Figures 1(b) and 1(c)). These images also demonstrated the proximity of the lesion with the femoral neurovascular bundle anteriorly and the obturator neurovascular bundle and the urinary bladder posteriorly. To avoid these important structures, a retrograde transpubic approach was planned to perform the procedure (Figure 1(d)).

**Figure 2:**
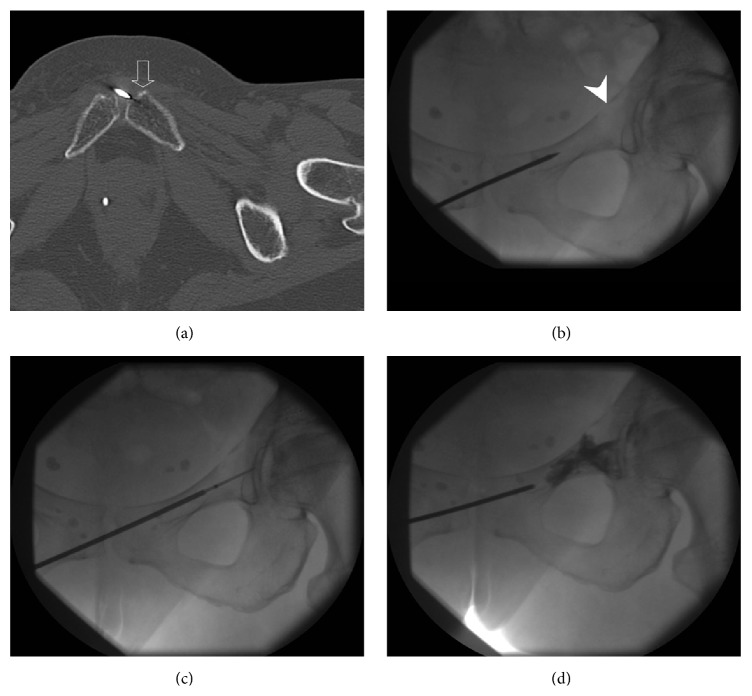
(a) Nonenhanced axial CT image demonstrates the entry point of the retrograde transpubic approach via the pubic tubercle (open arrow). (b)–(d) Anteroposterior fluoroscopic images show the needle guided through the superior pubic ramus toward the lesion (arrowhead) (b), the radiofrequency probe inserted coaxially (c), and the distribution of cement (d) at the end of the procedure.

The patient was positioned supine on the CT-scanner table, with the hip and knee joints in neutral position. Under local anesthesia and conscious sedation, with CT-fluoroscopic guidance, a 13-gauge Osteo-Site bone biopsy needle (*Cook Inc.*,* Bloomington*,* USA*) was introduced from the controlateral right side of the pubic symphysis and advanced into the ipsilateral left pubic tubercle (Figure 2(a)). The trocar was then oriented superiorly and laterally and advanced under fluoroscopic guidance and intermittent CT fluoroscopy through the left superior pubic ramus next to the target acetabular lesion (Figure 2(b)). A 17-gauge RF bipolar cooled, 20 mm active tip probe was inserted coaxially via the introducer (Figure 2(c)) and one RF current cycle was applied for 10 minutes with OsteoCool RF Ablation System (*Baylis Medical*,* Montreal*,* Canada*). The target temperature was recorded between 70° and 80°C. After removing the RF probe from the trocar, 4 mL of polymethyl methacrylate cement SpinePlex (*Stryker*,* Kalamazoo*,* USA*) was injected using the Precision Cement Delivery (PCD) system (*Stryker*) (Figure 2(d)). The immediate postprocedural CT-fluoroscopic control images demonstrated satisfactory result with no injury to the adjacent structures. There was no leakage of cement into neither the hip joint nor the surrounding soft tissue.

**Figure 3:**
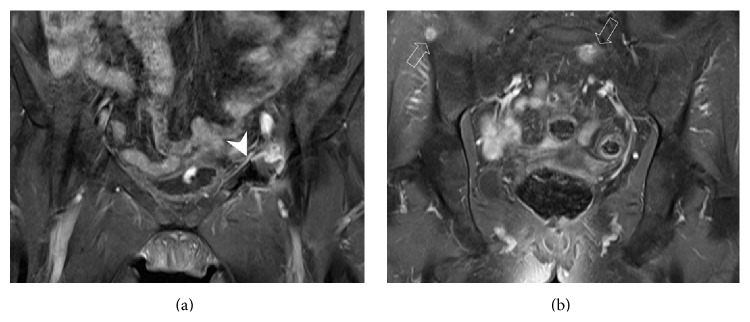
Coronal contrast-enhanced fat-saturated T1W images obtained two months after the procedure demonstrate complete tumor necrosis with thin peripheral rim enhancement of the ablation zone (arrowhead) but progression of the metastatic disease with two new pelvic lesions (open arrows).

One month following the procedure, the patient was seen by her oncology physician and showed mild clinical improvement as her pain was reasonably controlled (Visual Analogue Scale score 60/100 mm) and she was able to walk with a cane. Two months after the procedure, a follow-up MR study depicted the absence of recurrence or fracture of the treated lesion (Figure 3(a)) but progression of the metastatic bone disease in other areas of the pelvis (Figure 3(b)). Unfortunately, the patient died three months later from her metastatic disease without declared complications related to the percutaneous treatment.

## 3. Discussion

Percutaneous image-guided treatment of bone metastasis is now increasingly used, particularly for symptomatic acetabular lesions [8]. An anterior direct approach is routinely used to biopsy and treat lesions involving the anterior acetabular column [9]. However, some lesions cannot be accessed this way because of a complex anatomy or the proximity of pelvic visceral organs and neurovascular structures. With the aim of achieving satisfactory results and minimal risks of complications in our mind, we planned and performed a retrograde transpubic approach for radiofrequency ablation and cementoplasty of the anterior acetabular metastasis. To the best of our knowledge, this transpubic approach has not been reported in the radiological literature yet and was reputed very challenging in the orthopaedic literature. The development of retrograde transpubic screw fixation can be attributed to Lambotte as early as 1913 when he proposed this approach but did not actually perform the procedure [10]. The transpubic approach for intramedullary screw fixation was also described by Routt et al. [11] as an alternative to standard plating techniques for pubic ramus fracture fixation but exposed to vascular, neurologic, or visceral complications caused by inaccurate screw placement [11, 12].

Preprocedural planning is essential for a successful treatment with good outcome and no complications. Before proceeding to the RTP approach, radiologic assessment of the target region by CT scanning with bone and soft tissue windowing and multiplanar reformation is essential to evaluate the pubic ramus geometry, to identify the neighbouring vital structures, and to determine the entry point and target [7]. With this approach, it is important to choose a skin entry point controlateral to the pubic symphysis aligned with the long axis of the superior ramus. Additionally, the use of a bevelled needle allows applying forces for orienting the needle and can influence on the distribution of cement [13].

We prefer the dual guidance technique using CT and C-arm fluoroscopy when performing percutaneous tumor ablation and cementoplasty. This technique is safer since it provides a precise identification of the needle path through the pubic ramus and allows a well-controlled cement injection [14]. While the CT is helpful for needle guidance in the axial plane (*x*- and* y*-axes), the C-arm fluoroscopy allows guidance and control in a craniocaudal direction (*z*-axis) as well as real-time imaging during cement injection. The CT is also useful to prevent injury to adjacent soft tissue and organs structures, not seen on fluoroscopy. Suzuki et al. [7] stated that percutaneous screw fixation in a small area such as a pubic ramus or acetabulum is difficult to perform under fluoroscopy, but computer-assisted surgery can achieve a smaller margin of error [7, 15]. However, it is more cumbersome and time-consuming than using C-arm fluoroscopy alone [13]. Interestingly, the state-of-the-art recent flat-panel fluoroscopy systems with rotational acquisitions may nowadays replace the association of CT-scan and C-arm fluoroscopy, thanks to their ability to combine imaging in all three directions and to obtain reconstructed axial images together with a built-in navigation system [16]. Although slightly less precise than the dual guidance system, flat-panel systems deliver less radiation dose and reduce the procedure time [16, 17]. Further prospective studies are required to assess its feasibility and performance in comparison to the dual guidance technique.

In summary, we have described a successful combined palliative treatment with bipolar RF ablation and cementoplasty of an anterior acetabular osteolytic metastasis using a retrograde transpubic approach. This approach provides a safe access avoiding injury to noble structures close to the target lesion that might occurred otherwise with a direct anterior approach.
